# The differences between the subtypes of obstructive sleep apnea among different provinces in Turkey: a multicenter study

**DOI:** 10.1007/s00405-025-09271-6

**Published:** 2025-02-20

**Authors:** Sidika Deniz Yalim, Nazan Bayram, Imran Ozdemir, Cemal Cingi, Nuray Bayar Muluk

**Affiliations:** 1 Department of Otorhinolaryngology, University of Health Sciences, Adana City Training and Research Hospital, Adana, Turkey; 2 Department of Pulmonary Diseases, Gaziantep University, Gaziantep, Turkey; 3 Department of Pulmonary Diseases, Uskudar University, Istanbul, Turkey; 4 Department of Otorhinolaryngology, Eskisehir University, Eskisehir, Turkey; 5 Department of Otorhinolaryngology, Kirikkale University, Kirikkale, Turkey

**Keywords:** Obstructive sleep apnea syndrome, Polysomnography, Apnea-hypopnea index, Sleep stages, Oxygen

## Abstract

**Purpose:**

Our aim in this study is to investigate the significance and implications of different clinical subtypes of patients with obstructive sleep apnea syndrome (OSAS) in three different sleep centers from varied provinces.

**Methods:**

Between January 2023 and November 2024, 330 patients from three sleep centers (Adana, Gaziantep, and Istanbul) were retrospectively examined for age, gender, body mass index (BMI), polysomnography parameters, sleep stages, arousals, oxygen parameters, heart rates, apnea-hypopnea indexes (AHI) and snoring.

**Results:**

OSAS patients of Gaziantep were fat and severe OSAS patients who slept lightly but efficiently at lower oxygen levels with longer durations. They fell into Rapid Eye Movement (REM) sleep quickly. The number of hypopneas is high. OSAS patients of Adana sleep deeply, frequently wake after sleep onset, and snore less. The number of obstructive apnea is high. OSAS patients of Istanbul sleep efficiently but snore a lot. REM oxygen saturation, oxygen desaturation index (ODI) levels, and the average heart rate were high in Istanbul. The number of central and mixed apneas is high.

**Conclusions:**

Sleep patterns and oxygen measurements varied among clinical subtypes of patients with OSAS, as well as among provinces. Understanding regional or subtype-specific OSAS could alter practice because treatment can be planned according to the severity of OSAS.

## Introduction

Obstructive sleep apnea syndrome (OSAS) is a partial or complete collapse of the airway while sleeping causing apnea and hypopnea. Apnea is a reduction in airflow of ≥ 90% for at least 10 s. Hypopnea is defined as a drop in oxygen saturation of ≥ 3% or arousal lasting at least 10 s, compared to baseline. The apnea-hypopnea index (AHI) is the average number of apneas and hypopneas per hour of sleep [1].

OSAS is defined by at least 5 obstructive apneas, hypopneas, or both per hour throughout the patient’s sleep. OSAS is commonly divided into 3 levels of severity: mild (AHI = ≥ 5 but < 15 events per hour); moderate (AHI = 15–30 events per hour); and severe (AHI = > 30 events per hour) [2].

Nightly polysomnography in a sleep laboratory is the most successful test identifying obstructive sleep apnea, with the apnea-hypopnea index as its main measure. This test necessitates continuous monitoring of both sleep and breathing. Electroencephalograms, electro-oculograms, and chin electromyograms are used to assess cycles of sleep and wakefulness. Breathe reports should comprise estimates of respiration diligence, inflow tracking by nasal airflow, and blood oxygen concentration monitoring via a heated air detector. Electromyography of the proximal tibia is also commonly performed to evaluate limb motions [3].

Normal sleep has two primary stages: non-REM (Rapid Eye Movement) (NREM) and REM. Non-REM sleep is divided into three stages: N1, N2, and N3 sleep stages. The N1 sleep stage is the light sleep phase, a transitional time between deeper sleep stages during which an individual can easily be roused. The N2 sleep stage is deeper sleep, accounting for over half of human sleep time, while the N3 sleep stage is the deepest sleep. REM sleep is a peculiar sleep state in which the brain is engaged, muscle tone is greatly lowered, and respiratory and cardiac events, as well as dreams, are the most common [4].

Total sleep time (TST) is the amount of time spent sleeping and is a reliable indication of health. Sleep efficiency (SE) is calculated by dividing the time spent sleeping by the time spent in bed; normal values are 85% or above. Wake after sleep onset (WASO) refers to the total number of minutes a person remains awake after falling asleep. Sleep latency refers to the amount of time it takes to fall asleep. REM latency is the period between the onset of sleep and the first REM stage [5].

The oxygen desaturation index (ODI) is the number of times oxygen levels drop throughout an hour of sleep. T90 is the fraction of total sleep time with an oxygen saturation of less than 90%, indicating the duration and severity of hypoxia during sleep [6].

There are three types of sleep apnea; central, obstructive, and complex. Obstructive apnea occurs when breathing continues despite airflow interruption. Apnea is deemed to be of central origin if there is no contemporaneous respiratory effort. Mixed apnea is a combination of these two conditions. We commonly see obstructive sleep apnea. Central sleep apnea and complex sleep apnea are very different entities, therefore in this study, we will compare three distinct subtypes of OSAS; classic OSAS, positional obstructive sleep apnea syndrome (POSAS), and REM-related sleep apnea syndrome [7].

REM-related obstructive sleep apnea is a subtype of OSAS defined by apneas or hypopneas that occur mostly during REM sleep and is a neurologic issue. POSAS is a subtype of OSAS in which sleep-related obstructive episodes appear mostly in the supine position [8].

The treatment of the classic OSAS is continuous positive airway pressure (CPAP) or bilevel positive airway pressure (BPAP). However, the handling of REM-related OSAS and POSAS is automatic positive airway pressure (APAP). As a result, diagnosis of various forms of OSAS is crucial.

According to the definition of the American Academy of Sleep Medicine (AASM) classic OSAS was diagnosed by observing 5 or more obstructive apneas per hour on polysomnography. The commonly used diagnosis of REM-related OSAS is made when the ratio of the AHI value during REM sleep to the AHI value during NREM sleep (REM AHI/NREM AHI) is at least 2. POSAS is diagnosed with an AHI that is at least 2 times higher than that of a non-supine position (Table 1).

**Table 1:** OSA subtypes and their diagnostic criteria

OSA subtype	Diagnostic criteria
Classic OSA	5 or more obstructive apneas per hour on polysomnography
REM-related OSA	Ratio of the AHI value during REM sleep to the AHI value during NREM sleep (REM AHI/NREM AHI) is 2 or more
Positional OSA	AHI that is at least 2 times higher than that of a non-supine position

We aimed to investigate whether there are differences between different clinical subtypes of patients with obstructive sleep apnea syndrome in three different sleep centers from varied provinces to assess a large cohort of patients. To reflect wider regional variations, we selected Istanbul from the highest-populated region, Adana from the middle-populated region, and Gaziantep from the lowest-populated region of our country.

## Materials and methods

### Data collection

Between January 1, 2023 and November 2024 330 patients, 235 men, 95 women, (range 14–74 years) from three sleep centers (Adana, Istanbul and Gaziantep) from different provinces were retrospectively examined for the age, gender, body mass index (BMI), total recording time (TRT), total sleep time (TST), N1 sleep stage, N2 sleep stage, N3 sleep stage, REM sleep duration, wake after sleep onset (WASO), sleep period time, sleep latency, REM latency, sleep efficiency (SE), respiratory arousals, respiratory arousal index, spontaneous arousals, spontaneous arousal index, the total number of arousals, total arousal index, mean oxygen saturation, NREM oxygen saturation, REM oxygen saturation, minimum oxygen saturation, oxygen desaturation index (ODI), awake oxygen saturation, the time below 90% oxygen saturation (T90), mean heart rate, minimum heart rate, maximum heart rate, the number of obstructive, central and mixed apneas and hypopneas, mean apnea duration, NREM apnea-hypopnea index (AHI), REM AHI, AHI, total snoring events and snoring index.

All of the patients included were previously diagnosed with OSAS. Both OSAS and the normal control group patients had polysomnography for one night in the sleep laboratory. The patients with comorbidities and confounders (cardiovascular disease, smoking, and medication use) were excluded from the study because they affect the quality of sleep. Also, those who refused to take the tests, and those with incomplete records were excluded from the study group.

Polysomnography tests were performed using Comet Grass devices (Astro-Med, Inc., West Warwick, Rhode Island, United States) in Adana, Alice 6 (Philips Respironics, Murrysville, PA, United States) in Gaziantep, and, Neuron-spectrum-5 (Neurosoft, Ivanova, Russia) in Istanbul and scored according to the AASM guidelines.

### Statistical analysis

IBM Spss statistics 27.0 (IBM Corp., Armonk, NY, USA) software was used to analyze the data obtained in this study. In all analyses, the significance level was accepted as *p* < 0.01 and this was adjusted for multiple comparisons. Continuous variables are expressed as mean + standard deviation or median, and categorical variables are expressed as frequency and percentage. The Chi-square test and Fisher’s exact test were used when comparing categorical variables between groups. Since the sample size varies among groups, sensitivity analysis was made. Shapiro-Wilk test was applied to evaluate the normality distribution of the data. In cases where the normality assumption was not met, the differences between groups were examined using the Kruskal-Wallis H test. When significant results were obtained, pairwise comparisons between groups were made with the Mann-Whithey U test with Bonferroni correction. One-way ANOVA test was used for variables that met the normality assumption, and the Tukey post-hoc test was performed for pairwise comparisons between groups when significant differences were detected. To account for confounders (BMI, age, and gender) multivariate analyses were performed.

## Results

### Adana participants

In the Adana study group, which consisted of 30 classic, 30 positional, 30 REM-related OSAS, and 30 control group patients, the average age of the control group was lower than the other groups. There was no discernible variation between BMI and gender (Table 2).

**Table 2:** Adana participants’ demographics and values

	Clasic OSA	REM OSA	Positional OSA	Control	*p*
Age^†^	45.90 ± 8.77	51.03 ± 13.51	46.77 ± 9.56	41.13 ± 9.29	0.005*
WASO^†^	132.86 ± 94.50	92.54 ± 65.62	70.95 ± 59.44	83.90 ± 54.65	**0.006**
Respiratory Arousal^¶^	37.5(9-227)	19(0-158)	0(0-512)	2.5(0-182)	**< 0.001** ^**^
Arousal Index^¶^	6.5(1.5–48.4)	3.25(0-36.5)	0(0-122)	0.4(0-34.2)	< 0.001**
Spontan Arousal^¶^	75(0-189)	58(0-159)	0(0-209)	55(0-181)	**< 0.001** ^**^
Spontan Arousal Index^¶^	17.05(0-50.3)	11.4(0-31.5)	0(0–69)	11.1(0-101.3)	**0.001** ^**^
Total Arousal^¶^	133.5(35–249)	97(36–197)	163.5(0-829)	63(0.2–191)	**< 0.001** ^**^
Total Arousal Index^¶^	23.1(6.3–57.6)	16.75(6.9–39)	36.7(9.2–221)	15.6(2.3–125)	**< 0.001** ^**^
Mean Oxyhemoglobin^¶^	93.2(83.4–95.7)	93.1(82-96.1)	93.5(10.4–96.1)	94.3(90.9–97)	**0.008** ^**^
NREM Sao2^¶^	92.9(83.3–95.5)	93(81.4–96)	93.45(26.7–95.9)	94.2(90.9–97.2)	**0.016** ^**^
Total minimum Sao2^¶^	74.5(50–90)	78(50–89)	82.5(11.2–92)	84(50–95)	**< 0.001** ^**^
ODI^†^	17.02 ± 15.59	9.08 ± 9.49	26.78 ± 23.14	0.58 ± 0.62	**< 0.001** ^*^
T90^¶^	34.9(3.5-345.6)	31.75(0.2-262.2)	14.35(0-234.6)	9.95(0-290.3)	**0.005** ^**^
Obstructive Apnea^†^	190.40 ± 116.80	112.13 ± 91.81	104.03 ± 131.52	9.13 ± 10.28	**< 0.001** ^*^
Central apnea^¶^	1(0–60)	2(0–14)	0(0–23)	0(0–4)	**0.001** ^**^
Mixed apnea^¶^	1(0–80)	1(0–14)	0(0–47)	0(0–1)	**< 0.001** ^**^
Hypopnea^†^	19.90 ± 38.60	8.93 ± 8.88	67.53 ± 63.24	1.17 ± 2.28	**< 0.001** ^*^
Mean apnea duration^†^	26.74 ± 7.95	23.05 ± 7.28	18.47 ± 6.27	19.05 ± 13.11	**0.002** ^*^
NREM AHI^¶^	41.65(9-90.2)	13.6(0.2–68.2)	23.95(4.7-127.4)	1.5(0-5.1)	**< 0.001**
REM AHI^†^	19.34 ± 25.80	46.69 ± 20.56	20.79 ± 21.59	1.80 ± 3.31	**< 0.001** ^*^
AHI^†^	40.17 ± 24.33	40.17 ± 24.33	30.59 ± 28.67	1.82 ± 1.48	**< 0.001** ^*^

^†Mean±Standard Deviation. ⁍n(%).¶Medyan(Min−Max). *p*<0.01. *One way ANOVA. **Kruskal−Wallis H Test^

The difference between WASOs (*p* = 0.0063) is statistically significant. WASO of the classic OSAS group was considerably higher than the other groups (*p* < 0.01). The respiratory arousal averages and the arousal index averages of the classic OSAS group were considerably higher than those of REM-related OSAS, POSAS, and the control groups (*p* < 0.01). Spontaneous arousal index averages were considerably higher in the classic OSAS group compared to the other three groups (*p* < 0.01) (Table 2).

The mean oxyhemoglobin, NREM oxygen saturation, lowest oxygen saturation mean and ODI values of the POSAS group were remarkably higher than both classic and REM-related OSAS groups (*p* < 0.01). Classic OSAS averages were higher than REM-related OSAS averages (*p* < 0.01). The T90 scores of the classic OSAS group were substantially higher than the POSAS and the T90 scores of the REM-related OSAS group were remarkably higher than the POSAS and control (*p* < 0.01) (Table 2).

In terms of the number of obstructive apneas, central apnea, and mixed apneas, the mean of the classic OSAS group was substantially higher than others (*p* < 0.01) In terms of hypopnea the POSAS group had substantially higher values (*p* < 0.01). Correspondingly apnea duration, the classic OSAS group had higher values than REM-related and control (*p* < 0.01). REM-related OSAS had absolutely higher NREM AHI values than the POSAS and control (*p* < 0.01) (Table 2).

### Istanbul participants

The Istanbul study group consisted of 30 POSAS, 30 classic OSAS, and 30 control group patients. The classic OSAS group had higher BMI values than the POSAS group (*p* < 0.01) (Table 3).

**Table 3:** Istanbul participants’ demographics and values

	Classic OSA	Positional OSA	Control	*p*
Total Recording Time^¶^	402.43 ± 18.26	412.77 ± 16.15	388.60 ± 19.03	< 0.001*
Total Sleep Time^†^	290.77 ± 50.79	339.80 ± 16.47	344.47 ± 31.33	< 0.001*
N2^†^	40.60 ± 8.20	37.56 ± 7.14	32.73 ± 6.26	< 0.001*
REM duration^†^	20.28 ± 4.69	21.53 ± 5.02	25.83 ± 5.31	< 0.001*
WASO^†^	27.48 ± 11.44	22.59 ± 10.94	58.65 ± 16.41	< 0.001*
Sleep period time^†^	402.43 ± 17.87	411.43 ± 14.61	389.77 ± 18.09	< 0.001*
Sleep latency^¶^	11.36 ± 6.39	10.23 ± 3.75	22.95 ± 9.64	< 0.001*
REM latency^†^	226.49 ± 50.03	200.42 ± 30.58	160.89 ± 46.47	< 0.001*
Sleep efficiency^†^	71.63 ± 12.00	81.60 ± 4.30	90.20 ± 4.41	< 0.001*
Obstructive Apnea^†^	117.03 ± 32.04	35.17 ± 15.86	7.87 ± 5.61	< 0.001*
Central apnea^¶^	24.87 ± 9.22	8.87 ± 5.95	1.53 ± 1.50	< 0.001*
Mixed apnea^¶^	40.27 ± 13.36	24.23 ± 13.72	2.03 ± 2.40	< 0.001*
Hypopnea^†^	97.63 ± 23.95	41.70 ± 13.08	3.17 ± 2.60	< 0.001*
Mean apnea duration^†^	48.94 ± 11.20	14.62 ± 3.87	1.47 ± 1.03	< 0.001*
NREM AHI^¶^	11.01 ± 5.56	4.26 ± 2.77	1.05 ± 1.02	< 0.001*
REM AHI^†^	59.91 ± 14.85	18.93 ± 6.15	2.50 ± 1.54	< 0.001*
AHI^†^	260.40 ± 64.57	94.03 ± 66.31	34.60 ± 27.64	< 0.001*
Total snoring^¶^	56.08 ± 17.04	16.16 ± 11.34	5.91 ± 4.50	< 0.001*
Total snoring index^¶^	117.03 ± 32.04	35.17 ± 15.86	7.87 ± 5.61	< 0.001*

^†Mean±Standard Deviation. ⁍n(%).¶Medyan(Min−Max). *p*<0.01. *One way ANOVA. **Kruskal−Wallis H Test^

Total recording time, total sleep time, N2 sleep stage, REM sleep duration, and WASO were absolutely higher in the classic OSAS than in the POSAS group (*p* < 0.01). REM latency of the classic OSAS group was higher than the POSAS group (*p* < 0.01). The sleep period time and sleep efficiency of the classic OSAS group were absolutely lower than the POSAS group (*p* < 0.01) (Table 3).

Although oxyhemoglobin measurements were visibly lower than in the control group, they were lower in classic OSAS than in POSAS (*p* < 0.01). Classic OSAS NREM oxygen saturation levels were visibly lower than the POSAS group (*p* < 0.01). The classic OSAS group had considerably higher mean OSAS scores than the POSAS group (*p* < 0.01) (Table 3).

The classic OSAS group had considerably higher obstructive apnea, central apnea, mixed apnea, and hypopnea scores than the POSAS group. The classic OSAS group outperformed the POSAS group in terms of NREM AHI, REM AHI, and total AHIs, whereas the control group had the lowest values (*p* < 0.001) (Table 3).

The classic OSAS group had considerably higher snoring durations and indexes than the POSAS group, while the control group had lower values (*p* < 0.001) (Table 3).

### Gaziantep participants

The Gaziantep study group consisted of 36 classic OSAS, 33 REM-related OSAS, 35 POSAS, and 16 control group patients. Based on the analysis results, the average ages, BMI, and gender of the classic OSAS, REM-related OSAS, POSAS, and control groups differ statistically significantly (*p* < 0.001). It was determined that men had lower rates of REM-related OSAS, and this difference was statistically significant (Table 4).

**Table 4:** Gaziantep participants’ demographics and values

	Clasic OSA	REM OSA	Positional OSA	Control	*p*
Age^†^	45 ± 11.74	46.73 ± 12.36	48.94 ± 10.52	36.94 ± 11.53	0.008*
BMI^†^	34.4 ± 6.64	33.64 ± 8.08	33.23 ± 6.11	25.12 ± 6.64	< 0.001*
Gender^⁍^					
Male	28(77.8)	16(48.5)	31(88.6)	8(50)	< 0.001*
Female	8(22.2)	17(51.5)	4(11.4)	8(50)	
N1^¶^	48.5(16.5–171)	23(6–64)	42(16-105.5)	15.25(8.5–47.5)	< 0.001**
N3^†^	55.1 ± 46.05	70.64 ± 27.59	51.95 ± 28.07	85.48 ± 28.52	0.004*
REM latency^†^	159.±113.33	112.55 ± 79.98	146.64 ± 76.27	195.19 ± 93.18	0.025*
Respiratory Arousal^¶^	198 ± 156.91	32.27 ± 28.82	84.89 ± 88.40	6.25 ± 6.35	**< 0.001** ^*^
Arousal Index^¶^	34.1(0.5-106.3)	5.1(0-21.9)	11.9(0.4–702)	0.6(0-3.9)	**< 0.001** ^**^
Total Arousal^¶^	248 ± 152.09	79.00 ± 45.53	133.60 ± 95.07	66.38 ± 34.93	**< 0.001** ^*^
Total Arousal Index^¶^	49.7 ± 30.50	14.92 ± 7.82	26.25 ± 18.79	13.34 ± 7.21	**< 0.001** ^*^
Mean Oxyhemoglobin^¶^	89(71.5–96.5)	91(80–95)	91.5(9.5–94.5)	94.25(89–97)	**< 0.001** ^**^
NREM Sao2^¶^	89.0 ± 4.17	91.00 ± 2.46	91.06 ± 2.68	94.13 ± 1.93	**< 0.001** ^*^
REM Sao2^¶^	88(64–97)	91(76–95)	91(67–95)	94.5(89–97)	**< 0.001** ^**^
Total minimum Sao2^¶^	68.8 ± 14.24	79.09 ± 7.01	78.74 ± 9.35	89.31 ± 3.93	**< 0.001** ^*^
ODI^†^	70.3 ± 41.47	19.69 ± 12.72	39.17 ± 23.18	3.35 ± 5.14	**< 0.001** ^*^
Wakefulness Sao2^¶^	91.5 ± 2.66	92.15 ± 2.55	92.23 ± 2.00	94.69 ± 1.49	**< 0.001** ^*^
T90^¶^	128 ± 99.70	99.28 ± 106.87	90.09 ± 94.63	10.24 ± 39.38	**< 0.001** ^*^
Maximum heart rate^†^	101 ± 11.62	93.21 ± 10.61	98.03 ± 11.39	96.13 ± 14.69	**0.043** ^*^
Obstructive Apnea^†^	116.5(0-523)	2(0–71)	11(0-433)	0(0–1)	**< 0.001** ^**^
Central apnea^¶^	1.5(0–73)	1(0–14)	2(0–76)	0(0–5)	**0.012** ^**^
Mixed apnea^¶^	1(0-252)	0(0–7)	0(0–81)	0(0–2)	**0.008** ^**^
Hypopnea^†^	141.5(25–636)	70(10–259)	158(15–385)	13.5(2–22)	**< 0.001** ^**^
Mean apnea duration^†^	18.6 ± 5.53	14.75 ± 3.92	17.68 ± 5.12	17.75 ± 5.66	**0.019** ^*^
NREM AHI^¶^	75.8 ± 40.75	14.95 ± 10.65	52.18 ± 43.75	2.45 ± 1.27	**< 0.001** ^*^
REM AHI^†^	52.6 ± 40.23	52.13 ± 27.81	34.13 ± 25.41	5.31 ± 7.81	**< 0.001** ^*^
AHI^†^	72.0 ± 39.83	20.86 ± 12.39	41.26 ± 23.68	2.82 ± 1.28	**< 0.001** ^*^
Total snoring^¶^	81.0 ± 45.37	37.43 ± 31.48	79.68 ± 31.86	6.71 ± 9.22	**< 0.001** ^*^
Total snoring index^¶^	67.8 ± 40.16	15.34 ± 13.63	20.30 ± 15.14	1.80 ± 1.32	**< 0.001** ^*^

^†Mean±Standard Deviation. ⁍n(%).¶Medyan(Min−Max). *p*<0.01. *One way ANOVA. **Kruskal−Wallis H Test^

N1 sleep stage was totally longer than the control group being longest in the classic OSAS group. The classic OSAS group showed considerably higher overall arousal and arousal index values than other groups.

It was shown that the classic OSAS group had the lowest NREM and REM oxygen saturation and minimum oxygen saturation levels. The classic OSAS group had the highest ODI. The POSAS group had completely lower ODI values than the REM-related OSAS group (*p* < 0.001) (Table 4).

The classic OSAS group had lower T90 values than the positional and REM-related OSAS groups (*p* < 0.05). The classic OSAS group had considerably greater peak heart rate values than the REM-related OSAS group (Table 4).

The classic OSAS group had noticeably greater obstructive apnea rates than the other groups (*p* < 0.001). The classic OSAS group had higher values for central apnea, mixed apnea, and hypopnea (*p* < 0.01) (Table 4).

The classic OSAS group had considerably longer mean apnea duration (*p* < 0.01), higher NREM AHI values than the REM-related OSAS group (*p* < 0.01). The classic OSAS group exhibited considerably higher AHI values than REM-related OSAS, POSAS, and the control group (*p* < 0.01) (Table 4).

Regarding supine AHI, the classic OSAS group was found to have substantially higher values ​​than the REM-related OSAS and control groups (*p* < 0.01). The classic OSAS group had substantially higher lateral AHI values than the REM-related OSAS, POSAS, and control groups (*p* < 0.01) (Table 4).

### Comparison between centers

There was no significant difference between the Adana, Istanbul, and Gaziantep study groups regarding age (*p* = 0.49). However significant differences were detected between the groups regarding BMI (*p* < 0.01). In particular, it was observed that the BMI average of the Gaziantep study group was positively higher than the Adana and Istanbul groups, and the Adana study group had positively higher BMI averages than the Istanbul group (*p* < 0.01). In terms of gender distribution, the differences between groups were found to be statistically significant.

**Fig. 1:**
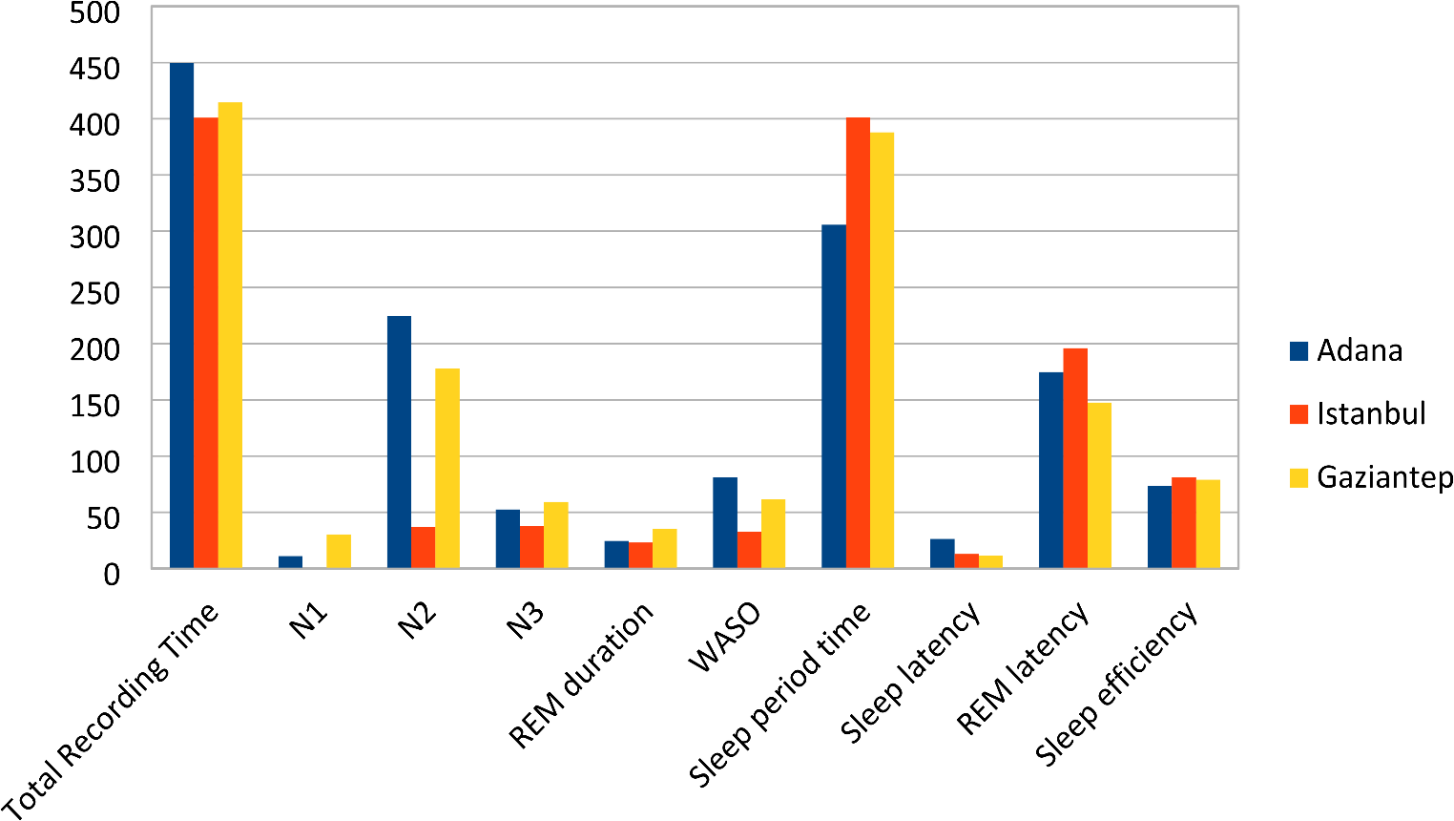
Comparison chart of the sleep patterns of the three sleep centers

The total sleep recording time of the Adana group was totally higher than that of Istanbul and Gaziantep (*p* < 0.01). However, there was no significant difference between the total sleep times of the three groups (*p* = 0.383). N1 sleep stage was found to be higher in the Antep group compared to Istanbul and Adana groups. The N2 sleep stage was higher in the Adana group compared to Istanbul and (*p* < 0.01). N3 sleep stage and REM sleep duration were completely lower in the Istanbul group compared to Adana and Gaziantep (*p* < 0.01) (Fig. 1).

WASO averages were highest in the Adana group and the averages of the Gaziantep group were significantly higher than those in Istanbul (*p* < 0.01). The sleep period of the Adana group was noticeably shorter than the Istanbul and Gaziantep groups (*p* < 0.01). The sleep latency average of the Adana group was significantly higher than the Istanbul and Gaziantep groups (*p* < 0.01) (Fig. 1).

The REM latency of the Gaziantep group was absolutely shorter than that of the Adana group, but it was absolutely longer than that of the Istanbul group (*p* < 0.01). The sleep efficiency of the Istanbul group was found to be totally higher than that of Adana (*p* < 0.01). The sleep efficiency of the Gaziantep group was totally higher than that of Adana (*p* < 0.01) (Fig. 1).

**Fig. 2:**
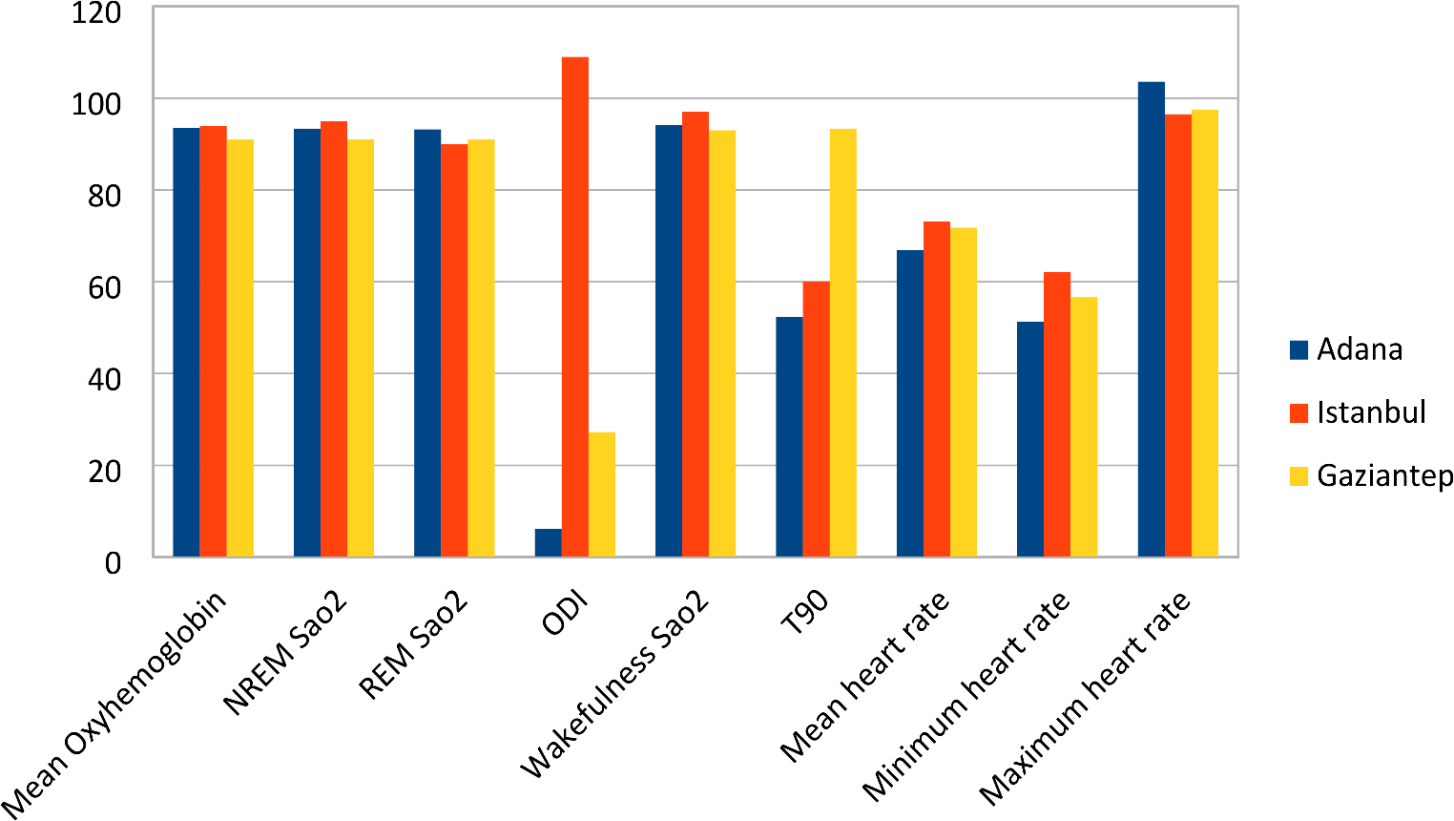
Comparison chart of the oxygen and cardiac parameters of the three sleep centers

The average oxyhemoglobin value of the Gaziantep group is substantially higher than both Adana and Istanbul (*p* < 0.01). The NREM oxygen saturation average of the Gaziantep group was substantially lower than Adana and Istanbul (*p* < 0.01). The mean of REM oxygen saturation was substatntially higher in the Istanbul group than in the Adana group (*p* < 0.01) (Fig. 2).

Istanbul had higher ODI values than Adana and Antep (*p* < 0.01). ODI values of Adana are visibly lower than Gaziantep (*p* < 0.01). In terms of awake oxygen levels Istanbul had higher values than Adana and Gaziantep but Gaziantep had lower awake oxygen saturation levels than Adana (*p* < 0.01) (Fig. 2).

Regarding T90 values Gaziantep showed higher values than Adana and Istanbul (*p* < 0.01). While Adana had lower values in average heart rates than Istanbul and Gaziantep, Gaziantep also had lower values than Istanbul (*p* < 0.01). Regarding the lowest heart rate, Adana had lower values than Istanbul and Gaziantep while Istanbul had higher values than Gaziantep (*p* < 0.01) (Fig. 2).

**Fig. 3:**
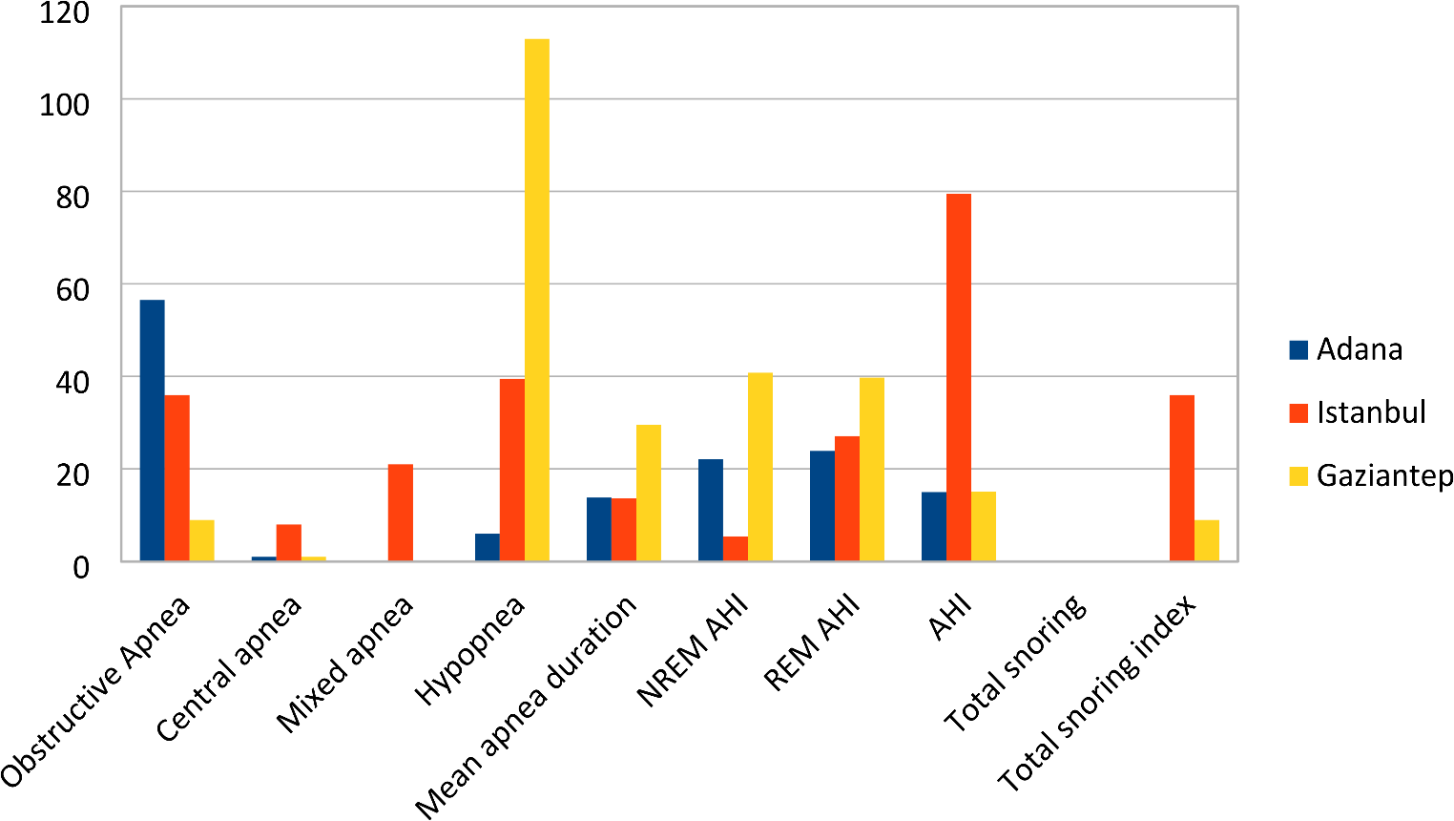
Comparison charts of the apneas, AHIs and snoring of the three sleep centers

Adana’s obstructive apnea scores were significantly higher than Istanbul and Gaziantep while Istanbul’s scores were higher than Gaziantep’s (*p* < 0.01). Central apnea scores of Adana and Gaziantep were lower than Istanbul (*p* < 0.01) but no significant difference was observed between Adana and Gaziantep. Mixed apnea scores were significantly higher in Istanbul than in Adana and Gaziantep (*p* < 0.01). Adana had lower hypopnea values than Gaziantep and Istanbul (*p* < 0.01). Gaziantep’s hypopnea values were higher than Istanbul’s (*p* < 0.01) (Fig. 3).

Gaziantep’s NREM AHI and REM AHI averages were higher than Adana’s and Istanbul’s (*p* < 0.01) Adana’s REM AHI was higher than Istanbul. Gaziantep had higher AHI values than Adana (*p* < 0.01) but there was no significant difference between Istanbul and Adana (Fig. 3).

Regarding total snoring events, Istanbul had totally higher averages than Adana and Gaziantep (*p* < 0.01). The total snoring index was totally lower in Adana compared to İstanbul and Gaziantep (*p* < 0.01) (Fig. 3).

## Discussion

When we look at the literature we found very few studies about the characteristics of OSAS subtypes and differences from different places. Our study is the first from our country to compare the subtypes of obstructive sleep apnea. We chose three cities from three varied regions of our country with the highest (Istanbul), lowest (Gaziantep), and medium (Adana) populations representing a broader regional difference. The findings will have clinical and social implications. The results will have social and clinical ramifications. It will provide us with a general understanding of the OSAS subtypes that are prevalent in that area. Also, culturally tailored evidence-based interventions that assess social determinants of health frameworks will reduce the burden of OSAS.

We found that the Adana group’s sleep was mostly short but deep sleep like normal people’s but with lower oxygen levels for long periods. Adana’s classic OSAS group woke up early shortly after falling asleep and very frequently and also had difficulty falling asleep. Obstructive, central, and mixed apnea scores were high in classic OSAS. However, the POSAS group woke up most frequently and had the highest oxygen levels. CPAP treatment is suitable for this group. POSAS group had higher oxygen parameters but high hypopnea scores.

In Istanbul’s classic OSAS group are fat and sleep light sleep mostly with low oxygen levels for long periods. Obstructive, central, mixed apneas, and hypopnea scores and OSAS intensity were higher in classic OSAS. This group benefits from weight loss and CPAP.

In Gaziantep men had lower rates of REM-related OSAS. Deepest sleep reduced the most. The classic OSAS group woke up frequently. They had long light sleep and sleep at low oxygen levels with longer periods. Obstructive, central, mixed apneas, and hypopnea scores were higher. Their OSAS intensity was higher. BMI of Gaziantep group was higher. This group benefits from weight loss and CPAP or BPAP.

There were no differences between the TSTs. The N1 sleep stage was high in Gaziantep, N2 high in Adana, and N3 low in Istanbul. WASO was highest in Adana. In Adana the sleep period was brief but the sleep latency was high. REM latency was shorter in Gaziantep. Sleep efficiency was higher in Istanbul and Gaziantep than in Adana. Gaziantep had a high mean oxyhemoglobin saturation and a decreased NREM oxygen saturation. REM oxygen saturation and ODI levels were high in Istanbul. T90 was elevated in Gaziantep. The average heart rate was high in Istanbul. Obstructive apnea prevalence was high in Adana. Central and mixed apnea incidences were high in Istanbul. The hypopnea rate was high in Gaziantep. NREM AHI, REM AHI, and total AHI were all greater in Gaziantep. Istanbul had a high incidence of snoring overall.

Sleep patterns and oxygen measurements varied among clinical subtypes of patients with obstructive sleep apnea syndrome, as well as among provinces. Classic OSAS mostly affected men. The classic OSAS group had a high intensity of OSAS. POSAS patients had less severe OSAS and nocturnal hypoxia.

**Fig. 4:**
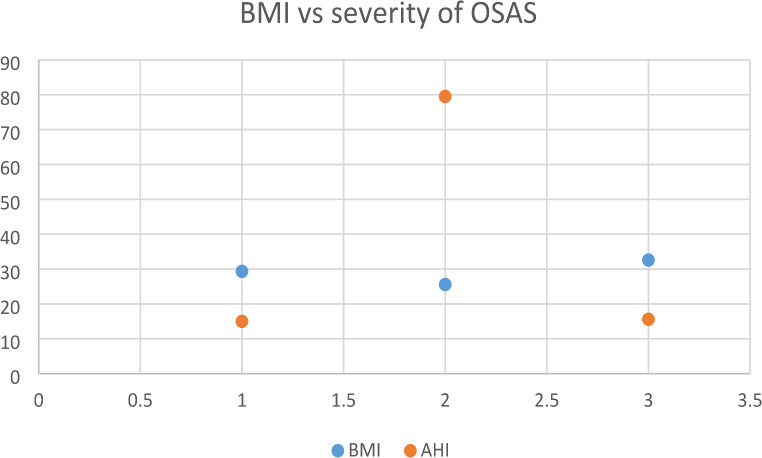
Scatter diagram of the BMI vs. severity of OSAS

Sunnetcioglu et al.compared the differences between REM-related OSAS and NREM OSAS and found that supine AHI had more effect than REM AHI on the severity of OSAS [9]. Similarly in our study both supine and side AHI were high in the classic OSAS group and the classic OSAS group had the high intensity (Fig. 4).

**Fig. 5:**
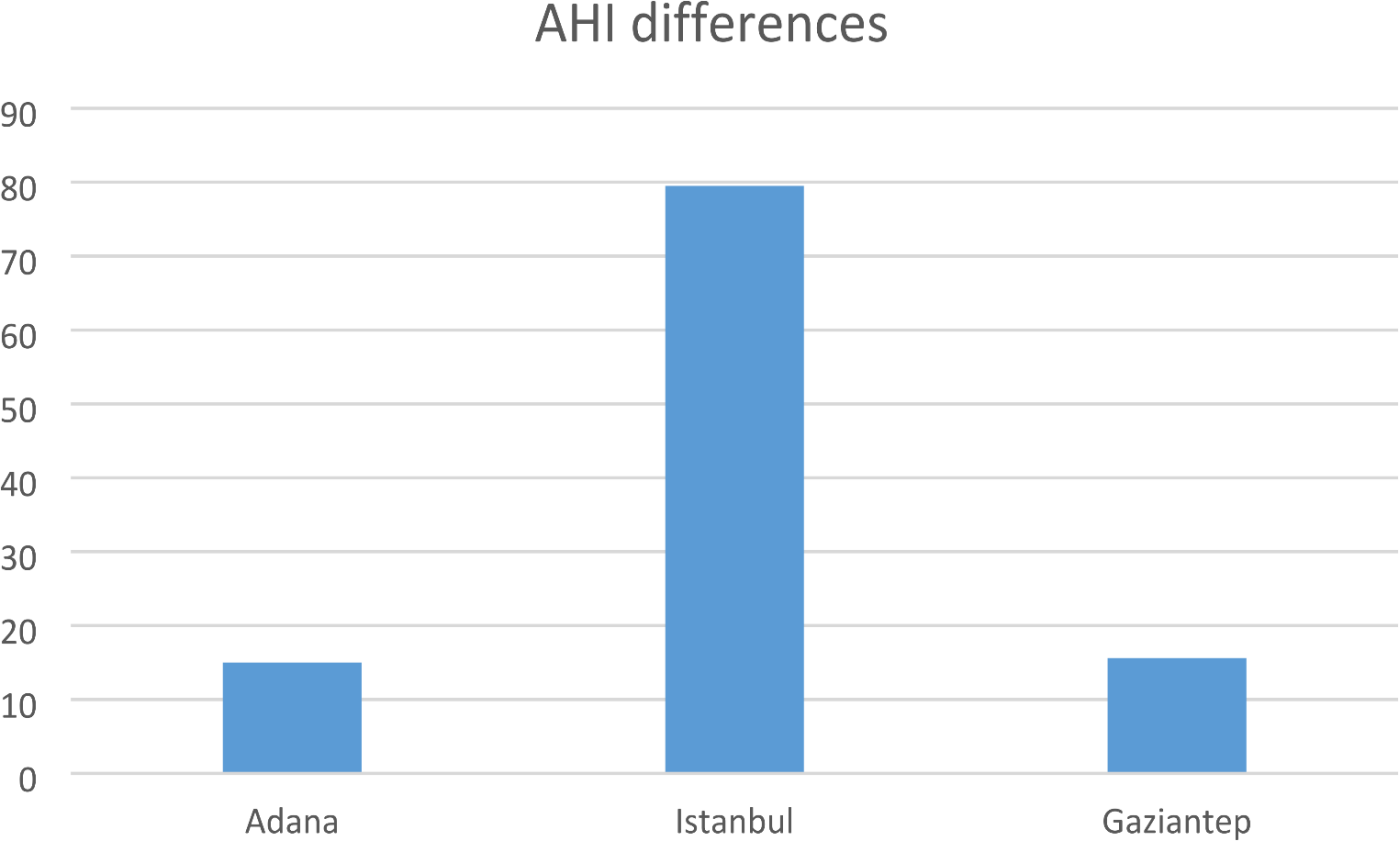
Bar chart of AHIs of the three sleep centers

Siddiqui et al. found that half of the patients with OSAS had a higher NREM AHI than REM AHI. Males dominated each group and there was no age difference between them. BMI and severity of oxygen desaturation were not absolutely different. Apneas were longer in the REM group. In our study males were significantly higher in classic OSAS and we found significant age differences but not in BMI (Fig. 5). Classic OSAS group had the high intensity. Similarly, REM-related OSAS had a longer apnea duration [10]. The differences between the results of the distinct studies may be due to methodological variations.

According to Koo et al., females have a higher prevalence of REM-related OSAS than males [11]. They found that REM-related sleep-disordered breathing prevalence falls with age in women as REM/NREM AHI possibly due to age-dependent rise in NREM/REM AHI. Obese young women were protected indicating age-related decline in female hormones [11]. We did not find a significant difference in terms of gender but in the Gaziantep group females had higher prevalence than men.

Sabil et al.’s large French population study with mild to moderate OSAS showed that POSAS was linked to time in the supine position, male gender, younger age, lower AHI, and lower BMI [12]. In our study consisting of mild, moderate, and severe OSAS patients, POSAS patients had less severe OSAS and nocturnal hypoxia and lower BMI.

In Huang et al.‘s study, approximately one-third of Chinese sufferers of OSAS had POSAS [13]. Chinese POSAS subjects had milder OSAS and nocturnal ischemia. Also, POSAS people had reduced arousal levels than non-POSAS participants. They stated that a low arousal limit could be an essential endotype in the etiology of POSAS, particularly in supine-isolated POSAS [13]. In our study, respiratory and spontaneous arousals and indices were higher in the classic OSAS but a total number of arousals and arousal index were higher in POSAS patients of the Adana group. These may be due to different endotypes of POSAS. The classic OSAS group had higher supine and side AHI values than the POSAS group. POSAS was found to have significantly higher supine AHI values ​​than the control group. The variations between the researchs’ results could be attributed to population heterogeneity.

Ida et al. classified Japanese OSAS patients in their single-center study and the classic OSAS cluster had been associated with younger adult age, obesity, and male preponderance [14]. In our study classic OSAS grouping’s mean age was higher than the control group. There were more men in the classic OSAS, but there was no change in BMI.

Ozdilekcan et al. discovered that individuals with a higher BMI experienced more frequent nocturnal oxygen desaturation spells, which led to higher arousal scores and decreased sleep efficiency [15]. REM-related OSAS with elevated BMI may increase nocturnal oxygen consumption [15]. In our study BMIs of OSAS groups were not significantly different but the classic OSAS group had lower oxygen desaturation levels and higher arousal thresholds than REM-related OSAS.

As reported by Zhang et al., REM-OSAS has a lower overall AHI than NREM-OSAS, but the length of apnea-hypopnea events is longer, and oxygen consumption is poorer [16]. Focusing solely on the overall AHI value will result in incorrect identification of REM-OSAS, and certain individuals having profound hypoxemia cannot be properly treated. REM-OSAS could be a precursor of OSAS, and active detection and prompt treatment could help patients. The etiology and mechanisms of REM-OSAS are currently unknown [16]. In our study REM-related OSAS had a lower overall AHI and oxygen parameters than classic OSAS and POSAS. However, the apnea duration of REM-related OSAS was shorter than classic OSAS but longer than the POSAS.

Potential mechanisms underlying the differences between subtypes of OSAS include genetic, cultural, and environmental. OSAS is prevalent in adults and children of Asian, especially Chinese and Japanese, Black and Hispanic/Latino origins. Neighborhood environment, the most studied social determinant of health partially explains racial/ethnic differences because OSAS is multifactorial. Environmental factors such as air pollution, passive smoking, occupational hazards, housing, job and food security, household income and wealth, access to sleep specialists, and sleep health literacy are influenced by differences in educational attainment [17].

The first limitation of our study is that we could have included more patients from more centers. Second, we had a limited number of women. We could have more female patients included. Finally, there may be potential biases due to the equipment differences between sleep laboratories, interpretation of polysomnography, and the lack of outcomes related to treatment.

## Conclusions

Sleep patterns and oxygen measurements varied among clinical subtypes of patients with obstructive sleep apnea syndrome, as well as among provinces. Understanding regional or subtype-specific OSAS could alter practice because treatment can be planned according to the severity of OSAS. For example, we may recommend oral appliance or surgical treatment in mild OSA, while we may recommend a PAP device in severe OSA. If we can describe the phenotypic subtypes and heterogeneity of OSAS, and if we can define ethnically diverse populations, in this field, we can be able to improve patient’s quality of life also benefit their families.

## Data Availability

Data supporting this study are not publicly available due to ethical and legal restrictions.
